# Building Memory Representations for Exemplar-Based Judgment: A Role for Ventral Precuneus

**DOI:** 10.3389/fnhum.2019.00228

**Published:** 2019-07-16

**Authors:** Sara Stillesjö, Lars Nyberg, Linnea Karlsson Wirebring

**Affiliations:** ^1^Department of Integrative Medical Biology, Umeå University, Umeå, Sweden; ^2^Umeå Center for Functional Brain Imaging, Umeå University, Umeå, Sweden; ^3^Department of Radiation Sciences, Umeå University, Umeå, Sweden; ^4^Department of Psychology, Umeå University, Umeå, Sweden

**Keywords:** multiple-cue judgment, exemplar-based model, cognitive modeling, fMRI, judgment and decision making, precuneus

## Abstract

The brain networks underlying human *multiple-cue judgment*, the judgment of a continuous criterion based on multiple cues, have been examined in a few recent studies, and the ventral precuneus has been found to be a key region. Specifically, activation differences in *ventral precuneus* (as measured with functional magnetic resonance imaging, fMRI) has been linked to an exemplar-based judgment process, where judgments are based on memory for previous similar cases. Ventral precuneus is implicated in various episodic memory processes, notably such that *increased* activity during learning in this region as well as in the ventromedial prefrontal cortex (vmPFC) and the medial temporal lobes (MTL) have been linked to retrieval success. The present study used fMRI during a multiple-cue judgment task to gain novel neurocognitive evidence informative for the link between learning-related activity changes in ventral precuneus and exemplar-based judgment. Participants (*N* = 27) spontaneously learned to make judgments during fMRI, in a multiple-cue judgment task specifically designed to induce exemplar-based processing. Contrasting brain activity during late learning to early learning revealed *higher* activity in ventral precuneus, the bilateral MTL, and the vmPFC. Activity in the ventral precuneus and the vmPFC was found to parametrically increase between each judgment event, and activity levels in the ventral precuneus predicted performance after learning. These results are interpreted such that the ventral precuneus supports the aspects of exemplar-based processes that are related to episodic memory, tentatively by building, storing, and being implicated in retrieving memory representations for judgment.

## Introduction

The act of multiple-cue judgment – to estimate a continuous criterion based on multiple cues – is something people engage in repeatedly in life. Consider for example how a teacher grades an essay, a car dealer estimates the price of a used car, or judges sentencing a criminal. Behavioral multiple-cue judgment research has traditionally focused on how people rely on rule-based processes, with weighted integration of each cue value, in order to make inferences. Linear regression models tend to describe such behavior well (e.g., Einhorn et al., 1979; Brehmer, 1994; Juslin et al., 2003). However, recent behavioral research has repeatedly demonstrated that judgments like these are often influenced by similarity-based comparisons to previously experienced similar situations stored in memory, a process that is well described by exemplar-based models (EBMs) (c.f. Medin and Schaffer, 1978; Nosofsky, 1984; see e.g., Juslin et al., 2003, 2008). It has even been suggested that similarity-based strategies are hard to resist when making inferences (Brooks and Hannah, 2006; Hahn et al., 2010; von Helversen et al., 2014a; Brumby and Hahn, 2017; Bröder et al., 2017).

Recently, this research was extended with brain imaging data demonstrating that the medial parietal cortex, specifically the ventral precuneus, might be key for a similarity-based judgment process (Wirebring et al., 2018). In that study, looking at brain activation in a test phase after learning, the ventral precuneus was activated compared to baseline in four different multiple-cue judgment conditions (*N* = 74), during both rule-based and similarity-based judgment processes. Interestingly, activity in this region predicted how well an EBM, but not a rule-based model, fit judgment data, raising the intriguing possibility that the role for ventral precuneus in human multiple-cue judgment is related to similarity-based processes (Wirebring et al., 2018).

Precuneus has received relatively little attention in the previous related literature, considering it might actually be a key hub for human judgment. Partly, this can be explained by the fact that several related studies in the field of categorization (i.e., the cognitively less demanding task where the criterion is binary instead of continuous) have focused on the logic of contrasting strategies against each other. Thus, to the extent that similarity-based processes are at play also when other strategies are executed, common activations in this brain region might have been canceled out. Similarity-based processes have instead by the logic of contrasting strategies for example been found to evoke higher activity than rule-based processes in the anterior prefrontal cortex and the inferior parietal cortex, whereas rule-based strategies instead evoke higher activity than similarity-based processes in the anterior cingulate cortex, the dorsolateral prefrontal cortex, and the posterior parietal cortex (e.g., Patalano et al., 2001; Grossman et al., 2002; Koenig et al., 2005; for reviews see also Nomura and Reber, 2008). Another reason for why precuneus might have been overlooked can be that several categorization studies have focused on *a priori* regions of interest, for example the medial temporal lobes (MTL). For instance, some recent studies have used model-based fMRI with EBMs and have found the MTL to be involved in concept learning (Mack et al., 2016), memory strength (Davis et al., 2014), and typicality of a memory representation used for exemplar-based similarity comparison (Davis and Poldrack, 2014). Others have detailed the neurocognitive processes involved in human category learning, without explicit reference to similarity-based processes *per se*, but with converging evidence for the involvement of the basal ganglia (Poldrack et al., 2001; Seger and Cincotta, 2005; Nomura et al., 2007; for a review see Seger and Miller, 2010) and cortico-striatal loops (e.g., Lopez-Paniagua and Seger, 2011). Finally, yet another reason precuneus have not been stressed in research on similarity-based judgment processes could be that judgment and categorization require different responses, where activity necessary for judgment might not necessarily be evoked in a categorization task, and vice versa. In one of the very few existing imaging studies of multiple-cue judgment, von Helversen et al. (2014b) demonstrated that making test-phase judgments with an exemplar-based strategy was associated with higher activity in the inferior parietal cortex and the inferior prefrontal cortex compared to a heuristic rule-based process, whereas a rule-based condition engaged the dorsolateral prefrontal cortex, precentral gyrus, and temporo-parietal regions more than an exemplar-based strategy. The reason that precuneus was not observed in that study could again be related to the possibility that the subtraction logic canceled out shared activity in precuneus. Notably, the ventral precuneus was recently found to be more engaged in an exemplar-based judgment condition compared to a rule-based judgment condition in a multiple-cue judgment task even though the conditions were directly contrasted (Wirebring et al., 2018) implying that experimental design might also be an important factor.

To the extent that ventral precuneus should be linked to exemplar-based processes, there are different possibilities pertaining to the role of this region in this type of process. One possibility is that the ventral precuneus is related to the *mnemonic representational* aspects necessary for exemplar-based processes in human multiple-cue judgment. The ventral precuneus has been extensively linked to episodic memory (Wagner et al., 2005; Vincent et al., 2006; Kaboodvand et al., 2018; for a review see Cavanna and Trimble, 2006) and has been found to re-activate for stimuli that are familiar due to recent exposure (the encoding-retrieval flip, for overviews see Huijbers et al., 2013; Gilmore et al., 2015). In addition, increased activity in the ventral precuneus, the MTL and the vmPFC during learning reflect mnemonic processing during encoding, and later retrieval success (e.g., Vilberg and Rugg, 2008; Daselaar et al., 2009; Hayama et al., 2012; Huijbers et al., 2013; Bonni et al., 2015). One hypothesis could thus be that the ventral precuneus in a similar manner could support exemplar-based judgment, with increased activity during learning as a function of more established memory representations used for judgment.

There are also other alternatives to how the link between the ventral precuneus and exemplar-based processes could be understood. For example, activity changes in precuneus could be related to *visuo-spatial attention* with the demands of attending to stimuli with multiple attributes (see e.g., for a review Cavanna and Trimble, 2006). Damage to this region has been shown to result in difficulties attending to a visual stimulus (Pflugshaupt et al., 2016) and has been reported when maintaining attention in an orientation discrimination task in healthy participants (Heinen et al., 2017). Moreover, the precuneus, including the ventral areas, routinely exhibits reduced activity over time in perceptually attention demanding cognitive tasks together with the vmPFC (e.g., Gusnard and Raichle, 2001; McKiernan et al., 2003). Thus, an alternative hypothesis could be that the ventral precuneus exhibit reduced activation during learning related to decreased attentional demands of performing a multiple-cue judgment task.

In the present study we aimed to use event-related functional magnetic resonance imaging (fMRI) during judgment learning to investigate how ventral precuneus plays a role in exemplar-based processes and whether this role should be characterized as reflecting mnemonic representational aspects, or visuo-spatial attention. If an exemplar-based process in the ventral precuneus is key for human judgment, we should see changes in this region during the learning phase. If so, we hypothesized that, if the ventral precuneus is involved in building memory representations for exemplar-based judgment, an *increased* BOLD signal should be observed in a number of *a priori* regions of interest: the ventral precuneus, the MTL and the vmPFC, during the course of learning. Higher activity in the ventral precuneus toward the end of learning was also expected to be directly related to performance on the learning items after learning, connecting activity levels to retrieval success (e.g., Daselaar et al., 2009; Hayama et al., 2012; for reviews see Vilberg and Rugg, 2008; Huijbers et al., 2013; Gilmore et al., 2015). On the other hand, if the ventral precuneus is rather involved in directing attention toward the different features of stimuli with multiple attributes, we hypothesized that a *reduction* of activity should be observed in the ventral precuneus during the course of learning (see e.g., Gusnard and Raichle, 2001; McKiernan et al., 2003) as a function of the task being less and less attentionally demanding. Finally, if brain activity in this region was in addition observed to be significantly higher than baseline across all scanned blocks, and this activity was functionally related to the fit of an EBM after learning, this would provide further support the suggestion that ventral precuneus is a key brain region multiple-cue judgment, and exemplar-based processes specifically (Wirebring et al., 2018).

During scanning, participants (young and healthy) spontaneously learned a multiple-cue judgment task based on a biological scenario. A multiplicative relationship between cue and criterion was applied, successfully used to induce exemplar-based processes in past behavioral studies (e.g., Juslin et al., 2008; von Helversen and Rieskamp, 2009) and two imaging studies (von Helversen et al., 2014b; Wirebring et al., 2018). It has been demonstrated that when it is difficult to rely on a linear weighted rule, for example when the cue-combination rule is multiplicative, and people spontaneously adhere to an exemplar-based strategy (see e.g., Juslin et al., 2008; Wirebring et al., 2018).

The experimental set up was designed to yield large differences in cognitive model predictions on the final test phase judgments between rule-based and EBMs, in order to confirm that the task manipulation described above had been successful. Some items were introduced during learning (training items) and a set of new items at test, and the behavioral responses were used to verify the models predictions. An EBM predicts that stored learning items will be retrieved from memory to make a judgment of new test items, with better performance for old than for new items and an inability to extrapolate to criterion values outside the acquired learning range (DeLosh et al., 1997; Juslin et al., 2003). A linear regression model on the other hand, where each cue is expected to be considered with a weighted linear rule, make no such predictions. The predicted differences in behavioral response, and the better model fit on test-phase data, was used to verify that participants spontaneously adopted an exemplar-based process in this task (see e.g., Karlsson et al., 2007, 2008; Juslin et al., 2008).

The critical comparison with regards to the brain imaging data concerned BOLD signal differences between late and early learning. Further characterization of activity changes in the brain regions for which we had *a priori* predictions (i.e., the ventral precuneus, MTL, and the vmPFC) included three *post hoc* tests to explicitly test for changes as a function of learning, and a correlation test to establish a link between the ventral precuneus activity and performance on training items (see section “Materials and Methods” for details).

## Materials and Methods

### Participants

A total of 34 participants (17 females; *M*_*age*_ = 24.9; Range = 20–38 years, *SD*_*age*_ = 4.54) were recruited at Umeå University through public advertisement. Participants were right-handed by self-report, met the criteria for MR-scanning (no metallic implants, not pregnant), were neurologically healthy, had no dyslexia or dyscalculia, and had not participated in previous similar studies. All participants provided written informed consent in accordance with the declaration of Helsinki. All procedures were approved by the Regional Ethical Review Board in Umeå, Sweden. Participants received 500 SEK for participation in the experiment. An extra bonus of maximally 200 SEK could be earned based on performance (*M*_*Bonus*_ = 124.9 SEK*, SD*_*Bonus*_ = 31.29 SEK).

Data from seven participants had to be discarded from all analyses: one participant did not reach the learning criterion, and six participants failed to complete all phases of the experiment. One additional participant was discarded from the brain imaging analyses because model fit of final test-phase data was numerically better by the rule-based model than by the EBM (see section “Results” for details).

### Design and Materials

In a within-subjects design, the task was to learn to judge the toxicity of fictional “tropical death-bugs” on a pseudo-continuous scale with outcome feedback, a task used in several previous studies on multiple-cue judgment to study rule-based and exemplar-based strategies (see e.g., Juslin et al., 2003, 2008; Olsson et al., 2006; Karlsson et al., 2007). The toxicity (the criterion) ranged between 2 and 28. The death-bugs differed on four binary cues (i.e., body parts) that affected the toxicity; the legs (long or short), the eyes (big or small), the head (dotted or striped) and the body (blue or purple).

To induce spontaneous adoption of an exemplar-based judgment strategy, we applied a task manipulation previously successfully used to make participants spontaneously learn to perform multiple-cue judgments with an exemplar-based strategy over a rule-based strategy (e.g., Karlsson et al., 2007; Juslin et al., 2008; Hoffmann et al., 2014; von Helversen et al., 2014b; Wirebring et al., 2018). It has repeatedly been shown that when a linear rule cannot be applied, as for example with a multiplicative relationship between cue and criterion, people tend to rely on exemplar-based strategies instead (Karlsson et al., 2007; Juslin et al., 2008; Wirebring et al., 2018). To relate to this work, the cue-combination rule used to calculate the toxicity was a multiplicative function of the cues:

(1) $$c=2+2.5\times e^{\left(4\times C_{1}+3\times C_{2}+2\times C_{3}+1\times C_{4}\right)/21.5}$$

where each cue, *C*_1_*, C*_2_*, C*_3_, and *C*_4_ could take a cue-value of −1 or 1 (see Table 1). For example, a death bug with the cue values (−1, −1, −1, −1) had a toxicity of 2, and a death-bug with the cue-weights (1, 1, 1, 1) had a toxicity of 28 (see Table 1). The cue weights, as well as which binary feature of the death-bug that implied toxicity, were randomly assigned for each participant.

**TABLE 1:** Structure of the task used in the experiment.

	**Cue values**						
**Subspecies**	**C1**	**C2**	**C3**	**C4**	**Toxicity**	**Task Set**	**Label**
1	−1	−1	−1	−1	2	New (intermediate)	I
2	−1	−1	−1	1	2	Training	T
3	−1	−1	1	−1	3	Training	T
4	−1	−1	1	1	3	New (intermediate)	I
5	−1	1	−1	−1	3	Training (intermediate)	TI
6	−1	1	−1	1	4	New (final test)	N
7	−1	1	1	−1	5	Training (intermediate)	TI
8	−1	1	1	1	6	New (intermediate)	I
9	1	−1	−1	−1	4	Training (intermediate)	TI
10	1	−1	−1	1	5	Training	T
11	1	−1	1	−1	6	New (intermediate)	I
12	1	−1	1	1	8	Training	T
13	1	1	−1	−1	8	Training (intermediate)	TI
14	1	1	−1	1	12	Training	T
15	1	1	1	−1	18	Training	T
16	1	1	1	1	28	New (final test)	N

*The table displays all subspecies of death bugs, cue combination (C1–C4), criterion level (toxicity) task set, and label. Items labeled T and T1 were used as training items, items labeled I and TI were used as intermediate test items and all items were used as final test items of which items labeled N were new for the final test.*

### Procedures

The experiment consisted of four sessions that were administered the same day. Participants began with a *familiarization session* outside the scanner where they were familiarized with the procedure for the fMRI judgment task and a few practice trials related to the cognitive-perceptual baseline task (dot-bug judgment task); judging whether a bug with similar resemblance to the death-bug had gray dots located on its body. The stimuli used in the familiarization session was unrelated to the main task in order to facilitate experience of the trial design and response mode but at the same time maintain novice to the to-be-learnt material before the start of the actual data collection. Participants were then informed about the *fMRI judgment session* where they should learn to judge the toxicity of a number of death-bugs with outcome feedback while being in the scanner. The death-bug judgment task was randomly alternated with the dot-bug judgment task (five items in each learning block) where participants were asked where on a bug dots were located; at the top, the bottom, or both. The structure of the dot-bug judgment task was identical to the death-bug judgment task on all aspects except that no outcome feedback was provided. Participants received no explicit instructions on how to solve the task.

Following the fMRI judgment session was a *behavioral judgment session*, including a *final test phase*, where participants continued to make judgments of death-bugs but outside of the scanner. Lastly, there was a *questionnaire session*, where participants answered questions regarding participants background, motivation, task difficulty, and knowledge on how the bodily features of the death bugs affected the toxicity.

#### fMRI Judgment Session

**FIGURE 1:**
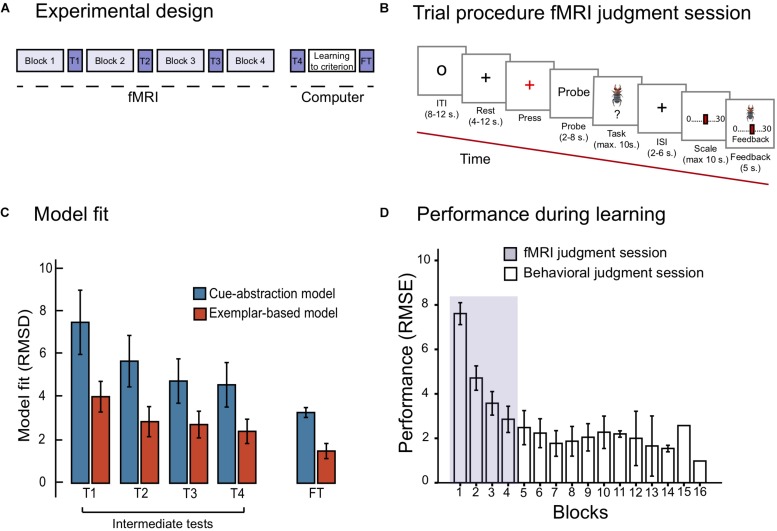
**(A)** Displays the experimental design, with the fMRI judgment session, the behavioral judgment session including the final test phase. **(B)** Displays the trial procedure during the learning blocks of the fMRI judgment session. **(C)** Displays model fit for the cue-abstraction model and exemplar-model on the intermediate tests (denoted T1, T2, T3, and T4) and the final test (denoted FT) reported in RMSD between participants judgment and model prediction. Note that a lower value on the *y*-axis reflects a better model fit. The blue bars display model fit to the cue-abstraction model. The red bars display model fit to the exemplar-based model (EBM). Error bars denote 95% CI around the mean. **(D)** displays performance reported in RMSE (root mean square error) between participants judgments and criteria to the learning criterion has been met (1.5 RMSE). The purple section displays RMSE on learning blocks performed during the fMRI judgment session. White bars display RMSE on learning blocks performed during the behavioral judgment session. Error bars denote 95% CI around the mean. Note that the last two bars include one participant only.

The task was displayed on a 32 inches computer screen inside the scanner. The participants viewed the screen through a mirror attached to the head coil. E-prime 2.0 (Psychology Software Tools, Inc., United States) was used to control stimuli presentation and logging of responses. A Lumitouch fMRI optical response four button keypad (Photon Control Inc., Canada) was used to collect responses. Participants entered the scanner where they learned to estimate the toxicity of the items from the training set (see Table 1) with outcome feedback of the correct numerical response. The session consisted of four *learning blocks*, wherein each of the 10 training items (death-bug judgment task) were presented twice in random order, in addition to the dot-bug judgment task (one block totaled 25 number of trials). In between each learning block was an *intermediate test phase*, used for cognitive modeling. See Figures 1A,B for an illustration of the experimental design and the fMRI trial design during learning and intermediate test, respectively.

On each trial, participants first viewed a small circle in the middle of the screen (8–12 s, Figure 1B). A gray cross hair (rest) was then presented which after a while changed color from gray to red (after 4–12 s). Participants were told to press the key with their right ring finger to indicate that they identified the color change. A probe with information text about the coming task was presented on the screen, either *death-bug* or *dot-bug* (2–8 s). Next, the stimuli to-be-judged (either a pictorial death bug or a dot bug) was presented for a maximum of 10 s. Participants were asked to make a judgment while the stimuli were displayed on the screen: either judging the toxicity of the displayed death-bug, or determining where dots were located, and press a button when they had formed their judgment. A jittered cross hair appeared when the button had been pressed or, if no button was pressed, after 10 s, and stayed on the screen for 2–6 s. That was followed by a scale where participants used their right-hand fingers to step to the location on the scale that matched their judgment. The scale was either ranging from 0 to 30 (for the death-bug trials) or with three alternatives (for the dot-bug trials; bottom, top or both). When a scale confirmation button had been pressed, or after 10 s, the pictorial death-bug became visible on the screen again together with the participants response and the correct numerical criterion as feedback (for 5 s). This was followed by the next trial (see Figure 1B).

One intermediate test was administered in between each of the four learning blocks that was scanned, yielding a total of four intermediate tests. In each intermediate test, four training items and four new items (denoted New Intermediate in Table 1) were judged twice in random order without feedback (yielding a total of 16 trials). No brain image acquisition was performed during the intermediate tests, and therefore no jittering was applied (see Figure 1B for test trial design).

#### Behavioral Judgment Session

The fourth intermediate test was administered immediately after the fMRI judgment session was completed, and took place in a room next to the scanner. After the fourth intermediate test, participants continued learning to judge the toxicity of the 10 training items until a learning criterion was met (RMSE between criterion and participants judgments ≤1.5 RMSE). The learning criterion was employed to ensure that all participants mastered the task about equally well at the end of learning.

After the criterion was met, there was a *final test phase*, where participants judged all training and all new items twice in random order without outcome feedback (see Table 1). Responses in the final test phase were used for cognitive modeling to confirm that participants had chosen to master the task with an exemplar-based strategy (see section “Quantitative Model Fit”). The trial design of the final test phase was identical to the intermediate test design (Figure 1B), but participants had 20 s (instead of ten) to complete the task. The purpose of the additional time was to maximize the quality of the data.

Lastly, participants answered a questionnaire with follow-up questions in the *questionnaire session* (results not reported here).

### Behavioral Data Analysis

#### Quantitative Model Fit

An EBM and a rule-based model (cue-abstraction model, CAM) were implemented in order to infer what strategy participants had relied on (see e.g., Juslin et al., 2003, 2008; Karlsson et al., 2007; Wirebring et al., 2018). The EBM assumes that judgment is based on the similarity of the to-be-judged stimuli and the memory of previously encountered exemplars (i.e., the training set). The numerical judgment ${\hat{C}}_{E\,B\,M}$ is determined by:

(2) $${\hat{C}}_{E\,B\,M}=\frac{\sum_{N}S_{n}.c_{n}}{\sum_{N}S_{n}}$$

where *N* is the previously encountered exemplars (10 in this particular experiment, see Table 1), *S*_*n*_ is the similarity between the to-be-judged item and the exemplar *n* and *c*_*n*_ the criterion value of exemplar *n*. The similarity-rule from the original context model (Medin and Schaffer, 1978) was used to calculate the similarity between a probe and a stored exemplar x_n_:

(3) $$S\,\left(n\right)=\prod_{i=1}^{I}d_{i}$$

where *d*_*i*_ represents an index of 1 if cue-values on cue-dimension *i* match (*i* = 1, …, I) and *s*_*i*_ [four free parameters in the interval (0,1)] if there is a mismatch.

With CAM it is assumed that the impact of each cue on the criterion is combined to form a judgment in a linear and additive way, captured by:

(4) $${\hat{C}}_{C\,A\,M}=k+\sum_{i=1}^{4}\omega_{i}.C_{i}$$

where the judgment ${\hat{C}}_{C\,A\,M}$ is a linear additive function of the cues *C*_i_.*k* (the intercept) and ω_i_ (the cue-weights) are free parameters (see Juslin et al., 2008).

The models’ were fitted to participants test-phase data using a *leave-one-out* cross validation procedure (Stone, 1974). For each participant, and for each of the five tests separately (the four intermediate tests and the final test), the models’ free parameters were estimated by fitting both models to all except one of the test items. A prediction for the remaining test item was rendered based on the estimated parameter values. This procedure was repeated for all test items. Unconstrained non-linear optimization with a *simplex* algorithm was used to estimate the parameters (MATLAB Inc., Natick, Mass). Goodness-of-fit was measured as the root mean squared deviation (RMSD) between the model’s predictions and the participant’s responses.

To confirm that participants adhered increasingly to an exemplar-based strategy over the course of the four scanned learning blocks, a 2 (CAM vs. EBM) × 4 (intermediate tests 1–4) repeated measurements ANOVA was done with RMSD of the EBM and the CAM, respectively as dependent variables. If the sphericity assumption was violated, corrected statistics using Greenhouse Geisser was applied. Finally, to confirm that participants were better fit by the EBM than the CAM during the final test phase, that is, after the learning criterion had been met, RMSD of the EBM, and the CAM was compared with a paired-samples *t*-test.

#### Performance

Performance was measured as root mean squared error (RMSE) between the criterion values and participants judgments on the death-bug task. To confirm that learning took place during the four blocks that were scanned with fMRI, RMSE of the four blocks (1–4) was entered into a repeated measurements ANOVA. If the sphericity assumption was violated, Greenhouse Geisser corrections were applied.

Differences in performance (RMSE) on training and new items during the final test phase was investigated with a paired samples *t*-test to accommodate the assumption of performance differences between items acquired during learning and novel test items (c.f. Juslin et al., 2003, 2008).

#### Extrapolation Index (EI)

To further confirm that the behavioral responses during the final test phase was in line with what can be expected by participants adhering to a similarity-based strategy, we calculated an *extrapolation index* (EI). EI was calculated by (5):

$$E\,I=\left\{ \begin{array}{c} x_{28}-p_{28}\\ p_{2}-x_{2} \end{array}\right.$$

where *x*_28_ and *x*_2_ are a participant’s response on items with the highest and lowest toxicity level, and *p*_28_ and *p*_2_ are the predicted judgments by linear extrapolation to the same items, where the prediction was based on regressing participants judgments of the ten training items against the criteria (see Juslin et al., 2008). A total of four deviations per participant (two repetitions of each of the two extreme test items during the final test) was considered, and averaged they constituted the *EI*. If *EI* = 0, this indicates perfect linear extrapolation, and is predicted by an optimal cue-abstraction model. A negative *EI* indicates poor extrapolation behavior and is predicted by the EBM.

### Neuroimaging Data Acquisition and Analysis

#### Image Acquisition and Preprocessing

Images were acquired using a 3.0 T whole body MRI system (MR 750, GE, Medical Systems) with an attached 32 channels head coil. A GE-EPI sequence with the following parameters was used: echo time (TE) 30 ms; repetition time (TR) 2000 ms; flip angle 80°; field of view (FOV) 25 cm; matrix = 96 × 96; 3.4 mm slice thickness (37 slices acquired). High-resolution T1-weighted structural images were obtained for each participant. The EPI sequence used for BOLD imaging was applied to acquire T2^*^-weighted images. Ten dummy-scans were performed to allow for equilibrium of the fMRI-signal before the start of the data collection. To minimize noise inside the scanner, subjects wore earplugs and headphones. Cushions were placed inside the head coil, one at each side, to minimize head movements. Functional data were preprocessed and analyzed using SPM12 (The Welcome Department of Cognitive Neurology, London, United Kingdom), implemented within an in-house program (*DataZ*). Images were corrected for slice-timing, head movement were corrected through unwarp, and realignment. Images were spatially normalized with Dartel (Ashburner, 2007), transformed to MNI-space, and smoothed (8 mm FWHM Gaussian filter kernel). Statistical analyses were calculated with smoothed data with a high-pass filter (128 s cutoff period) to remove low-frequency noise.

### General Linear Model Analyses

#### Changes in BOLD Signal Between Early and Late Learning

The primary analyses focused on regions with a different BOLD signal during late learning compared to early learning and was identified by comparing activity from the fourth (last) scanned learning block with the first scanned learning block (i.e., block 4 > 1) and vice versa (i.e., block 1 > 4).

In the first level analysis, individual general linear models (GLM) were estimated for each participant where the death-bug judgment task events and the dot-bug events during blocks one, two, three, and four were modeled as regressors of interest, and four regressors of no interest; rest (i.e., the cross hair, see Figure 1B) on all four blocks. The six movement parameters were included as covariates of no interest. All regressors except those associated with the head movements were convolved with a canonical hemodynamic response function. The duration of all events was modeled by using participants’ response time for each event. For example, the death-bug judgment task events and the dot-bug events were modeled from onset to button press, whereas the rest events were modeled from onset of the crosshair to button press. First level analyses consisted of contrasts comparing death-bug judgment task events from blocks one and four used to identify regions where BOLD signal was higher late compared to early during learning (i.e., block 4 > 1) and BOLD signal that was higher early compared to late during learning (block 1 > 4). Second-level analyses consisted of one sample *t*-tests based on the contrasts defined at the first-level, in a whole-brain analysis.

Three control analyses were performed to test the specificity of results obtained from the primary model for the contrast block 4 > 1. First, a model with the purpose to test if a tighter baseline comparison yielded results consistent with the primary model was defined. The five dot-bug judgment task events from the first learning block (block 1, early dot-bugs) and the five dot-bug judgment task events from the last learning block (block 4, late dot-bugs) were included as separate regressors in the model. A *t*-contrast [(death-bugs block 4 – dot-bugs block 4) > (death-bugs block 1 – dot-bugs block 1)] was defined at the first level, and evaluated at the second level in a one-sample *t*-test. Second, the difference between block 1 and 4 for the death-bug judgment task and the dot-bug judgment task [defined as (Task block 4 – Rest block 4) – (Task block 1 – Rest block 1)], respectively, was calculated. The purpose was to evaluate if the learning-related changes in the regions of interest were linked to the death-bug judgment task, and not the dot-bug judgment task, as evaluated with a paired samples *t*-test. Third, in order to investigate whether the effects would change by modeling additional events of no interest we defined a model where three additional regressors of no interest were included: the probe events, the scale events and the feedback events for all learning blocks (see Figure 1B). A *t*-contrast (block 4 > 1) was defined at the first level, and evaluated at the second level in a one-sample *t*-test. Note that both models in all other aspects were identical to the primary model.

The statistical threshold was set to *p* ≤ 0.05 (FDR corrected) at the voxel level and *k* > 0 at the cluster level for the whole brain analyses.

#### *Post hoc* Analyses

Next, a set of *post hoc* tests were performed to further characterize the activation pattern observed in the primary analysis where the activation map identified in the former step was used as mask to define our regions of interests (i.e., ventral precuneus, the vmPFC, and the MTL if activity increases, and ventral precuneus if activity was reduced).

First, block-wise comparisons were performed to test activity changes between each block (based on death-bug judgment task events >all dot-bug judgment task events), with paired-samples *t*-tests.

Second, to explicitly test whether there was a link between activity levels in the ventral precuneus and retrieval success (RMSE of old items at the final test, see items denoted T in Table 1), beta values extracted from the fourth learning block (5 mm radius around the peak voxel) was correlated with RMSE of training items at the final test.

Third, in two parametric analyses we tested for gradual changes between each judgment event (i.e., increases and reductions of the BOLD signal) that might be missed when averaging activity within blocks. The purpose of this approach was related to that repeated judgment continuously improve learning, such that more learning lead to better memory representations that can be retrieved for similarity comparison. First, exponential and linear adaption of the BOLD signal was evaluated separately, and the individual judgment task events (duration modeled as response time), and linear or exponential functions included as a separate regressor in the GLM (see Poldrack et al., 2001). BOLD signal that was affected by the modulator (i.e., displayed a linear or exponential increase in BOLD signal between each death-bug judgment task event) and two contrasts modeling these effects were defined. The six movement parameters were included as covariates of no interest. All regressors except the head movement parameters were convolved with a canonical hemodynamic response function. Effects were evaluated with second level one sample *t*-tests.

Statistical threshold for the post-hoc tests were set to *p* < 0.05 (Bonferroni corrected for number of regions, *p* < 0.05/4 = *p* < 0.016) and *k* > 0 at the cluster level.

#### BOLD-Signal Differences on the Judgment Task Collapsed Over All Scanned Blocks in Relation to Model Fit

Finally, it was evaluated whether ventral precuneus activity was significantly higher than baseline across all scanned blocks during death-bug judgments, and in addition if this activity was related to the fit of an EBM during the final test. Wirebring et al. (2018) recently demonstrated that activity in the ventral precuneus, when making judgments on novel test-phase items, predicted how well an EBM fit the judgment data. To evaluate which brain regions that were overall engaged during all learning blocks, a contrast collapsing all learning blocks, subtracted against the dot-bug judgment task, was defined (All blocks – All dot-bugs). A one-sample *t*-test at the second level was used to evaluate the contrast in a whole-brain analysis.

Finally, the activation map identified in the whole-brain analysis was again used as mask, and a one-sample *t*-test with the fit of the EBM was used as covariate of interest to test for a functional relationship between model fit and activity levels in the regions of interest that was consistently engaged during learning (ventral precuneus, vmPFC, and the bilateral MTL). Extracted beta values (5 mm radius around the peak voxel) was correlated with RMSD of the EBM on final judgment test-phase data.

## Results

### Behavioral Results

#### Quantitative Model Fit

With regard to the diagnostic comparison of model fit during the final test phase, the paired samples *t*-test did importantly confirm that EBM fit participants data better than CAM, *t* (26) = −11.21, *p* < 0.001 (see Figure 1C). However, one participant’s judgment data was found to have better model fit of CAM than EBM at the final test, and was therefore excluded from the imaging analyses.

The model fit during the four intermediate tests revealed that participants adhered increasingly to an exemplar-based strategy over the course of the four scanned learning blocks (see Figure 1C): a repeated measurements ANOVA with model fit (RMSD of EBM and CAM, respectively) and the four intermediate tests (1- 4) was performed. The ANOVA yielded a main effect of model [*F*_(1,26)_ = 100.91; *MSE* = 3.69; *p* < 0.001, $\eta_{p}^{2}$ = 0.79], and intermediate tests [*F*_(3,78)_ = 8.97; *MSE* = 8.46; *p* < 0.001, $\eta_{p}^{2}$ = 0.26]. There was no significant interaction between model and intermediate test [*F*_(3,78)_ = 2.36; *MSE* = 3.16; *p* = 0.094, $\eta_{p}^{2}$ = 0.83]. In sum, despite the fact that model fit of both models improved as a function of block, fit of the EBM was considerably better than the fit of CAM during all blocks (e.g., the fit of EBM was more than twice as good as the fit of CAM during the fourth intermediate test).

#### Performance During Learning

Performance (RMSE) as a function of blocks is shown in Figure 1D. Already during the first four blocks of learning (i.e., the blocks that were scanned) performance was drastically improved. A repeated measurements ANOVA on RMSE as a function of the first four blocks (1–4) was performed, reflecting performance during the fMRI judgment session. The ANOVA yielded a main effect of block [*F*_(3,78)_ = 99.67; *MSE* = 1.48; *p* < 0.001; = 0.79]. Paired samples *t*-tests moreover confirmed significant improvements between each of the first four blocks (all *ps* < 0.05). On average, participants reached the learning criterion (≤1.5 RMSE) after seven blocks (*M* = 7.4; *SD* = 3.03).

#### Performance During the Final Test

A paired samples *t*-test on RMSE demonstrated that participants were better at judging training exemplars compared to new exemplars in the final test phase, *M*_*old*_ = 1.31, *SD* = 1.1, *M*_*new*_ = 6.8, *SD* = 1.95*, t*(26) = −13.3*, p* < 0.001, a typical pattern predicted by EBMs (e.g., Juslin et al., 2003, 2008). This result hold even when excluding the most extreme extrapolation items with cue values (−1, −1, −1, −1) and (1, 1, 1, 1), *M*_*old*_ = 1.31, *SD* = 1.1, *M*_*new*_** = 2.2, *SD* = 1.31*, t*(26) = −2.48*, p* = 0.02.

On average, the *EI* based on judgments of the most extreme novel test items was negative and the confidence interval did not include zero [*M* = −3.84; 95% CI (−4.9, −2.79); *SD* = 2.67], demonstrating that participants were poor at extrapolating outside the acquired learning range. Again, this is a typical pattern predicted by EBMs (e.g., Juslin et al., 2003, 2008).

Taken together, the behavioral results suggest that participants overall learnt to master the task with an exemplar-based judgment strategy rather than adopting a rule-based judgment strategy.

### Imaging Results

#### Changes in BOLD Signal Between Early and Late Learning Blocks

**FIGURE 2:**
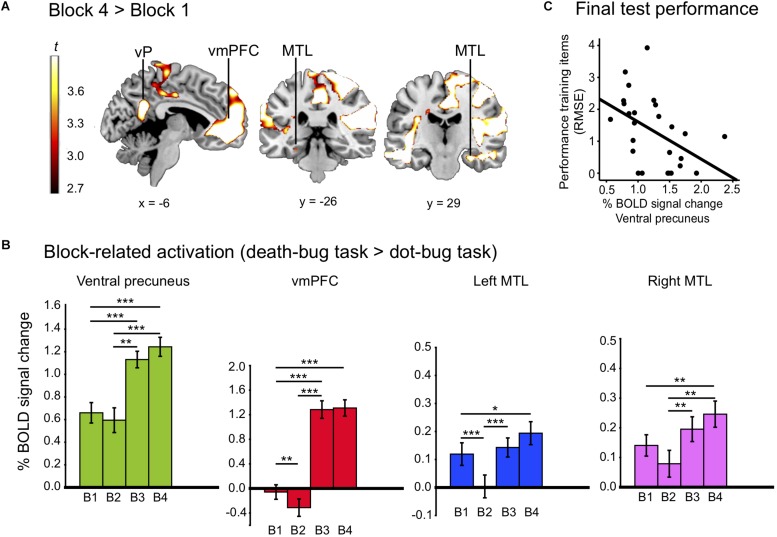
**(A)** Displays the activation map from regions that increased between learning blocks (block 4 > 1), including the ventral precuneus (vP), vmPFC, and the bilateral MTL. **(B)** Displays % bold signal change (*y*-axis) in the ventral precuneus, the vmPFC and the bilateral MTL for each learning block (B1–B4). **(C)** Display a correlation between activity drawn from block four in the ventral precuneus, and performance reported in RMSE of training items at the final test. The ^*^p < 0.05, ^∗∗^*p* < 0.01, and ^∗∗∗^*p* < 0.001.

If a similarity-based process in the ventral precuneus is key for human judgment, activity changes in the ventral precuneus was expected when learning to master a judgment task. The whole-brain analysis revealed changes between early and late learning (*p* < 0.05, FDR). The contrast (block 4 > 1) yielded effects in a number of large clusters that were more engaged during late learning than early learning (see Table 2 for localizations). Of most importance, we observed higher BOLD signal late compared to early learning in the ventral precuneus, the vmPFC, and the bilateral MTL. A number of other regions typically implicated in episodic memory retrieval was also identified, including the temporal pole and insula (see Figure 2A and Table 2 for localizations).

**TABLE 2:** BOLD-signal changes between early and late learning blocks.

	**Region**	**Hem**	**BA**	**MNI coordinates**			***t*-value**	**Voxels**
				***x***	***y***	***z***		
**Block four > Block one**								
1	Medial prefrontal	R	5/7	4	54	−6	8.67	6755
2	Supramarginal gyrus	R	2/48/20	70	−26	28	8.53	17526
	Middle temporal gyrus	R		54	−10	−20	7.6	
3	Rolandic operalis	L	20/21/23	−58	−66	2	7.59	8351
	Middle temporal gyrus	L		−56	−14	−18	7.1	
	Anterior cingulum	R		4	38	0	7.01	
	Ventral precuneus	L		−6	−54	26	5.97	
	Insula	L		−32	−24	24	4.95	
	Hippocampus	R		32	−18	−22	4.32	
4	Insula	R	48	36	6	14	4.02	42
5	Cerebellum	R		26	−80	−40	3.98	95
6	Superior frontal gyrus	L	8/9/32	−18	28	42	3.47	111
7	Parahippocampus	L	20	−28	−30	−18	3.21	23
8	Cerebellum	L		−24	−80	−40	3.03	14
	Temporal pole	R		34	12	−38	2.82	
9	Temporal pole	L	20/38	−34	16	−30	2.78	15
10	Precental gyrus	L	43	−54	−12	26	2.77	20
**Block one > Block four**								
1	Calcarine	R	18	12	−90	2	15.19	96108
	Anterior frontal gyrus	L		−34	52	10	11.76	
	Inferior parietal gyrus	R		42	−48	42	11.62	
	Superior frontal gyrus	L		−14	0	68	9.94	
	Caudate	R		12	4	12	5.66	
	Cerebellum	R		8	−76	−26	7.99	

*Hem, hemisphere; BA, Brodmann area. Coordinates (x, y, z) in MNI space (SPM12). Up to five local maxima are reported under each cluster. Only cluster 10 voxels or larger are reported. t-values at the peak voxel. Voxel: p < 0.05 (FDR corrected) cluster extension = 0.*

Paired sample post-hoc *t*-tests on block-related differences inside the mask revealed that there was a significant reduction in BOLD signal between the first and the second learning block in the vmPFC and the left MTL (all *p’s* < 0.05). Significant increases in the ventral precuneus, the vmPFC, and the bilateral MTL were observed between the second and the third block and the first and the fourth block (all *p‘s* < 0.05; see Figure 2B).

In support of the findings from the primary statistical model, all three control analyses yielded overlapping results. First, when contrasting the death-bug judgment task with the dot-bug judgment task, the group-level comparison [i.e., (block 4 – dot-bugs) vs. (block 1 – dot-bugs)] revealed an overlapping activation pattern, albeit under a less conservative statistical threshold (see Supplementary Figure 1A). Importantly, the pattern included BOLD signal differences in the ventral precuneus (*x, y, z* = −4, −54, 22; *t* = 3.05), the vmPFC (*x, y, z* = 6, 52, 10; *t* = 5.08), and the bilateral MTL (*x, y, z* = −26, −10, −28*; t* = 3.12*; x, y, z* = 32, −8, −28; *t* = 2.69). Second, the difference in BOLD activity between blocks 1 and 4 [defined by (Task4 – Rest) – (Task 1 – Rest)] was calculated for the death-bug judgment task and the dot-bug judgment task, respectively. The observed patterns in the regions of interest (ventral precuneus, vmPFC, and MTL) strongly suggested that the learning-related changes between block 1 and 4 were selective (or considerably stronger) in the death-bug judgment task than in the dot-bug judgment task (see Supplementary Figure 1B). Third, including additional regressors in the model yielded virtually identical results as in the primary model, again including the ventral precuneus (*x, y, z* = 8, −52, 22*; t* = 5.03).

**FIGURE 3:**
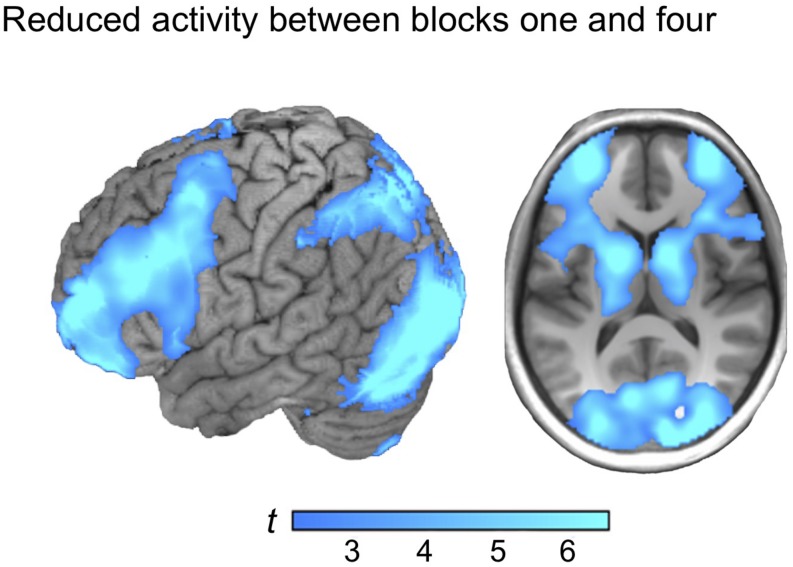
The activation map from regions that reduced between block one and block four (block 1 > 4).

Reversing the contrast (block 1 > 4) yielded marked BOLD signal differences in one large cluster, including frontoparietal regions and the basal ganglia, distributed over both hemispheres, with the strongest peak in the visual cortex (see Figure 3). Similar activation patterns have previously been observed in frontoparietal networks related to executive demands and cognitive control (see e.g., Vincent et al., 2008). Thus, it is plausible to observe reductions in such regions as a function of learning to master a task. No reduction of the BOLD signal in the ventral precuneus was observed under the chosen statistical threshold (see Table 2 for localizations).

#### Correlational Analysis Revealed a Link Between BOLD Signal in Ventral Precuneus and Performance on Training Items at the Final Test

As a next step, we investigated if a link between BOLD level activity in the ventral precuneus could be linked to the process of building memory representations. Correlating activity in ventral precuneus during the last scanned learning block (i.e., block 4) with performance on training items (RMSE) at the final test revealed a strong negative correlation [*r* (24) = −0.46, *p* = 0.019], suggesting that the higher the activity level in the ventral precuneus toward the end of learning, the better performance on those same training items during the final judgment test phase (see Figure 2C).

#### Parametric Analyses Confirmed Gradual Increase of the BOLD Signal in the Ventral Precuneus

The parametric method offered further insights into whether the BOLD signal gradually changed between each judgment event. First, parametric *increase* of the BOLD signal was evaluated inside the mask (block 4 > 1). Linear increase of the BOLD signal was observed in the ventral precuneus (*x, y, z* = 2, −62, 22; *t* = 3.89; *p* = 0.0003). Exponential increase of the BOLD signal was also identified in the ventral precuneus (*x, y, z* = 0, −64, 16; *t* = 2.80; *p* = 0.002) and in the vmPFC (*x, y, z* = −14, 74, 14; *t* = 3.35; *p* = 0.0012). No gradual changes were observed in the MTL under the chosen statistical threshold.

Parametric *reductions* were also evaluated using the activation map from the contrast (block 1 > 4) as mask. Linear reductions of the BOLD signal were observed, with the strongest peak in the left striatum including the head and body of the caudate (*x, y, z* = −20, 16, 8; *t* = 5.54; *p* < 0.001. The exponential parametric analysis also displayed a similar effect, with the strongest peak in the left putamen (*x, y, z* = −4, 4, −4; *t* = 4.22; *p* < 0.001) whereas no parametric reductions were observed in the ventral precuneus, the MTL or the vmPFC under the chosen statistical threshold.

#### A Link Between Exemplar-Based Model Fit and BOLD Signal in Ventral Precuneus During Judgment Learning

The whole-brain analysis collapsing all learning blocks against the perceptual baseline task (All blocks – Dot bugs) demonstrated effects in a large cluster covering both hemispheres, including prefrontal, temporal, and parietal regions. Notably, the strongest peak was located in the ventral precuneus in close proximity to the peak identified in Wirebring et al. (2018) and the cluster completely overlapped with the cluster identified in the contrast block 4 > 1 reported above (see Tables 2, 3).

**TABLE 3:** Overall BOLD-signal during learning.

**Region**	**Hem**	**BA**	**MNI coordinates**			***t*-value**	**Voxels (*k*)**
			***x***	***y***	***z***		
**All blocks vs. All dot-bugs**							
1. Precuneus	R	23	2	−64	22	20.09	93156
Middle occipital gyrus	L		−38	−88	−10	10.91	
Inferior occipital gyrus	L		−6	−96	6	10.06	
Superior frontal gyrus	L		−8	6	58	9.49	
Inferior frontal orbitalis	L		−22	30	−12	5.76	
Temporal pole	R		50	8	−12	5.76	
2. Cerebellum	R		12	−86	−44	4.31	204
3. Cerebellum	L		−10	−88	−44	3.45	43
4. Cerebellum	R		16	−72	−34	2.39	16

*Hem, hemisphere; BA, Brodmann area. Coordinates (x, y, z) in MNI space (SPM12). Up to five local maxima are reported under each cluster. Only cluster 10 voxels or larger are reported. t-values at the peak voxel. Voxel: p < 0.05 (FDR corrected) cluster extension = 0.*

In terms of a functional relationship between BOLD activity and model fit of the EBM during the final test, the results revealed negative correlations between BOLD level activity that was consistently engaged during learning and model fit of the EBM in the ventral precuneus [*x, y, z* = 12, −52, 10; *r* (24) = −0.52; *p* = 0.007] and also in the vmPFC [*x, y, z* = −18, 34, −12; *r* (24) = −0.46; *p* = 0.17] but not in the MTL.

## Discussion

Exemplar-based processes, as captured by EBMs, are important for adaptive human judgment, decision making, and categorization. The ventral precuneus was recently identified as a key brain region for human multiple-cue judgment and brain activity in this region could be linked to how well an EBM fit novel test-phase data (Wirebring et al., 2018). However, further research was needed in order to establish whether the role for this region in exemplar-based judgment processes was related to episodic memory, or rather to visual attention. In the present study we aimed to tackle this question by focusing on changes in BOLD signal over learning in a task where participants were learning to make judgments with an exemplar-based strategy while being scanned with fMRI. Participant’s judgment data at the final test was in line with behavioral predictions of an EBM. In addition, while minor decreases in RMSD as a function of learning is likely to partly reflect reduced measurement error related to the quality of the data as learning progressed, model fit of the four intermediate tests suggested that participants adhered increasingly to an exemplar-based strategy over the course of the four scanned learning blocks. This behavioral outcome was preceded by increased engagement of a large-scale network of brain regions previously demonstrated to be important for episodic memory processes, including the ventral precuneus, the vmPFC and the MTL (see e.g., Cabeza and Nyberg, 2000; Vincent et al., 2006; Rugg and Vilberg, 2013). Activity levels in the ventral precuneus predicted performance after learning. Moreover, brain activity in ventral precuneus was significantly higher than the perceptual baseline task even when collapsing across all learning blocks.

Neural correlates traditionally expected during episodic memory processes are frequently reported also in research on human judgment, decision making, and categorization (Patalano et al., 2001; Koenig et al., 2005; Volz et al., 2006; Milton et al., 2009; von Helversen et al., 2014b; Mack et al., 2016; Bowman and Zeithamova, 2018; Wirebring et al., 2018). For example, the MTL and the vmPFC have been found to be engaged during concept generalization (Bowman and Zeithamova, 2018), and the MTL has been linked to similarity-based processes in categorization (see e.g., Davis and Poldrack, 2014; Davis et al., 2014). The vmPFC has been implicated in response selection (for a review see Euston et al., 2012), value comparison, and confidence (e.g., De Martino et al., 2013) processes that are important for adaptive decision making. The ventral precuneus has also been reported in tasks expected to pose demands on episodic memory processes, for example when making judgments based on a recognition heuristic (Volz et al., 2006), and when categorizing stimuli based on similarity (Milton et al., 2009). Moreover, two studies on similarity-based categorization using quantitative measures of EBMs reported ventral precuneus in activation tables, in a task expected to pose demands on episodic memory (e.g., Mack et al., 2016; Bowman and Zeithamova, 2018). However, this is to our knowledge the first study that specifically target the potential role for the ventral precuneus in exemplar-based judgment processes.

How could the link between ventral precuneus and exemplar-based processes be understood? The ventral precuneus, together with the vmPFC and the MTL, has been linked to a network that enables episodic memory retrieval (e.g., Wagner et al., 2005; Vincent et al., 2006; Gilmore et al., 2015). It has been suggested that the ventral precuneus could act as a gateway between MTL and other cortical regions involved in episodic memory processes (Kaboodvand et al., 2018). The ventral precuneus could also be involved either in directing attention toward internal memory representations stored in the MTL, or in selection and retrieval of memory representations used for decision making (see Wagner et al., 2005). The precuneus was recently found to be a critical cortical node for coordinating MTL-cortical communication, and stimulation to this region altered memory vividness and ease of recall (Hebscher et al., 2019). Notably, the present study demonstrated that activity levels in the ventral precuneus, the vmPFC and the bilateral MTL increased between early and late learning blocks. Moreover, activity levels in the ventral precuneus toward the end of the learning phase was functionally related to performance at test, demonstrating a typical effect related to retrieval success (see e.g., Huijbers et al., 2013). Based on the present results, one possibility is that the ventral precuneus is involved in building and retrieving memory representations with the MTL and the vmPFC. Repetition yielded a stronger and more accessible memory representation that could be retrieved for exemplar-based judgment. The parametric increase in the ventral precuneus and the vmPFC, and the link between BOLD activity in the ventral precuneus and performance at the final test, could thus demonstrate a process of aiding retrieval, and response selection of stronger memory representations between trials. For example, exemplar-based processes in the vmPFC corroborates well recent findings linking this region to the level of evidence for similarity-based category decisions (Davis et al., 2017; O’Bryan et al., 2018). The ventral precuneus was consistently engaged during learning, and activity levels in the ventral precuneus and the vmPFC predicted EBM fit at the final test. This interpretation also connects the present findings to research on categorization focusing on the role of the MTL in building memory representations retrieved for similarity comparison (e.g., Davis et al., 2012; Davis and Poldrack, 2014; Mack et al., 2016).

It should be noted that the reduction of the BOLD signal between the first and the second learning block suggest that this process might not have been equally engaged during the entire fMRI judgment learning phase. Human judgment and decision making are known to be adaptive, with suggested contingent strategy shifts based on the content of the task (see e.g., Karlsson et al., 2007, 2008; Juslin et al., 2008; Bröder et al., 2010; Hoffmann et al., 2013; but see also Payne et al., 1993; Gigerenzer et al., 1999). One speculative interpretation of the specific reduction between block one and two could be that participants engaged in a different judgment strategy during the second learning block in order to establish the best strategy to solve the judgment task. However, it should also be noted that the parametric increase in the ventral precuneus and the vmPFC observed from trial to trial suggest that the process of building memory representations needed for exemplar-based judgment might continue at some level, irrespective of eventual strategy shifts.

In contrast to the robust increase between early and late learning blocks, reductions in activity in brain regions typically implied in a frontoparietal control system were observed between early and late learning (see e.g., Cole and Schneider, 2007; Dosenbach et al., 2007; Vincent et al., 2008; Zanto and Gazzaley, 2013). Related effects have been observed following extensive practice, and when people learn to cope with novel demands in an effortful task (e.g., Chein and Schneider, 2005; Kelly and Garavan, 2005). Importantly, we did not observe reduced activation in ventral precuneus as a function of learning under the chosen statistical threshold. The present results thus do not provide evidence for that the key role for ventral precuneus in multiple-cue judgment is related to demanding visuo-spatial attention processes that can be expected to be less demanding over time (see e.g., Gusnard and Raichle, 2001; McKiernan et al., 2003). It should be noted that one alternative interpretation of the present results could be that the task simply became easier with practice. If so, reduced activation in fronto-parietal regions, and engagement of the ventral precuneus, the vmPFC and the MTL could be a consequence of reduced effort. For example, nodes involved in attention and working memory demanding tasks are anticorrelated with deactivation in several brain regions during such processes, including default mode activation in the medial parietal cortex, the vmPFC, and the MTL (Fox et al., 2005). However, increased activity-levels in the MTL and the ventral precuneus over learning is well-established signatures for episodic learning (e.g., Gilmore et al., 2015). It has also been shown that gradual increases in the precuneus might be diagnostic for rapid creation of memory representations because this predicts successful behavior (Brodt et al., 2016). Thus, we find it more likely that the observed effect is not a mere consequence of effort, but rather reflects gradual accumulation of stored exemplar representations that guide behavior.

We did not have *a priori* predictions for all brain regions that exhibited changes over learning. Our choice to focus on the ventral precuneus, the vmPFC, and the MTL was based on the well-known relationship between activity levels in these regions and episodic memory processes (for reviews see e.g., Cabeza and Nyberg, 2000; Rugg and Vilberg, 2013; Gilmore et al., 2015). Nevertheless, future research could aim to specify how other brain regions reported in the present study are related to exemplar-based judgment learning to test the validity of our findings. Regions that could be of particular interest for future research on exemplar-based processes for human judgment is the insular cortex and the temporal pole. For example, the insular cortex has been suggested to be a hub for large-scale brain networks (for a review see Gogolla, 2017) and decision making, for example in action selection based on different alternatives (for a review see Droutman et al., 2015). The insula could in a similar manner support exemplar-based processes for human multiple-cue judgment, by its potential involvement in selection of exemplars used for similarity comparison. The temporal pole is believed to play an important role for memory retrieval of specific memory representations (see e.g., Binder et al., 2009; Timmers et al., 2011; Bonner and Price, 2013) and has been suggested to be an amodal hub that integrates information that is associated with a concept (Patterson et al., 2007).

Recently, a large-scale behavioral study where participants made multiple-cue judgments either with rule-based or exemplar-based strategies demonstrated that rule-based and exemplar-based processes draw on different cognitive resources, related to working memory and episodic memory, respectively (Hoffmann et al., 2014). The present results thus concur with the conclusions by Hoffmann et al. (2014) and further provide neurocognitive data informative for the link between exemplar-based judgment learning and episodic memory processes.

How do our results compare to detailed neurocognitive models of category learning? One of the most prominent models is the model COVIS (Competition between Verbal and Implicit Systems) suggesting that two competing neural systems underlie human categorization (Ashby et al., 1998). COVIS detail that rule-based processes are governed by the prefrontal cortex, and that implicit processes instead rely on the body and tail of the caudate. Recently, it was proposed that exemplar theory should be included in the procedural system of COVIS, where the caudate is linked to mediation of synaptic connections between the striatum and other cortical regions related to experience of the task at hand (Ashby and Rosedahl, 2017). Whereas some previous research in line with COVIS have identified gradual increase in the caudate (Poldrack et al., 2001; but see also Poldrack et al., 1999; Nomura et al., 2007), the present results rather demonstrated reductions in the caudate, both between blocks and parametrically between trials. One interpretation of the present results is that EBMs capture an episodic memory process governed by the ventral precuneus, the MTL and the vmPFC. However, it is possible that EBM fit in procedural learning tasks (e.g., information integration tasks) might correlate more with regions involved in procedural learning, such as the caudate. Exemplar-based processes could in that respect engage different neurocognitive processes depending on the content of the task, which sometimes might require episodic memory, and other times implicit memory. Future studies should be devoted to investigate the link between the caudate, human judgment and the EBMs‘ predictions to further clarify this topic.

### Limitations and Future Directions

One assumption in categorization (Ashby et al., 1998) and judgment (Juslin et al., 2008) is that people often try to identify relationships between cue and criterion when facing a novel task, a so-called rule-bias (e.g., Ashby et al., 1998; Juslin et al., 2008). However, the modeling results in the present study did not give support for a rule-bias; an EBM fit test-phase data better than a rule-based model on all intermediate tests. This result could imply that participants did not engage in initial hypothesis testing, but rather memorized exemplars immediately, supported also with the parametric increase in the ventral precuneus and the vmPFC. Similarity-based processes have been shown to be hard to resist (Brooks and Hannah, 2006; Hahn et al., 2010; von Helversen et al., 2014a; Brumby and Hahn, 2017; Bröder et al., 2017) and has been argued to be an unavoidable consequence of attending a stimulus in instance learning (Logan, 1988) which might suggest that an exemplar-based strategy was consistently used. Alternatively, the model fit measure used here is rather coarse, and fine-grained behavioral differences connected to a potential rule-bias within a learning block might have been missed. The observed reduction between the first and the second learning block might for example reflect a dynamic strategy shift related to a rule-bias that was not picked up with intermediate test-phase data. It should however, be stressed that the purpose of the present study was not to focus on dynamic strategy shifts during judgment learning. Further research could implement and test learning models of the assumed processes and relate measures from such models to fMRI data to gain more detailed information related to this question (see e.g., Davis et al., 2012, 2014; Mack et al., 2016).

One limitation of the present study is that it was not practically possible to scan the entire learning session, due to the fact that each trial in a multiple-cue judgment task require rather long events. Nevertheless, as was evident in the behavioral data, a large proportion of learning occurred during the first four learning blocks.

The dot-bug judgment task was included in the experiment to be able to control for sensory-motor activity related to inspecting the visual stimuli and conducting a response. This control task included a limited number of events (a total of 20 events, i.e., 5 per section of the experiment, block 1 – 4). The reasons for including fewer events in the dot-bug judgment task were (i) that the experiment was quite long, and (ii) no learning-related changes for the dot-bug judgment task with unique (not re-presented) items were expected. Still, ideally, the experiment should have included the same number of events (80) in both the death- and dot-bug judgment task. We note this as a limitation of the design, but highlight that the results pattern when the dot-bug events from blocks 1 and 4 were subtracted from the death-bug events indicated that learning-related changes were selective (or considerably stronger) in the death-bug judgment task.

Finally, the main purpose of the present study was to investigate how a similarity-based process in the ventral precuneus, critically suggested to be a key region for similarity-based and rule-based processes in human judgment (see Wirebring et al., 2018) should be understood. The present results suggest that the ventral precuneus is involved in a mnemonic process of building and retrieving memory representations used for similarity comparison in a task designed to capture exemplar-based processes specifically. The extent to which the ventral precuneus contribute to the same cognitive component process in rule-based judgment is unclear. The present results could guide future research to test whether an exemplar-based process in the ventral precuneus is involved also during rule-based judgment learning.

## Conclusion

We investigated how the ventral precuneus contribute to exemplar-based processes in human judgment. Focusing on differences in the BOLD signal between early and late learning during similarity-based judgment learning, we investigated implications for this region in mnemonic representational processes. The results showed an increase of the BOLD signal in a parietal memory network related to retrieval success, including the ventral precuneus. Moreover, activity in the ventral precuneus toward the end of learning predicted retrieval success after learning. Thus, our findings indicate that activity levels in the ventral precuneus reflect gradual accumulation of exemplars in memory that could guide behavior, presumably by building memory representations for exemplar-based judgments.

## Ethics Statement

This study was carried out in accordance with the recommendations of the Regional ethical Review board in Umeå, Sweden. All subjects gave written informed consent in accordance with the Declaration of Helsinki. The protocol was approved by the Regional ethical Review board in Umeå, Sweden.

## Author Contributions

All authors conceived and designed the study, wrote sections of the manuscript, and revised, read, and approved the submitted version of the manuscript. SS was responsible for data collection and wrote the first draft of the manuscript. SS and LKW performed the statistical analyses.

## Conflict of Interest Statement

The authors declare that the research was conducted in the absence of any commercial or financial relationships that could be construed as a potential conflict of interest.
