# Supplementary figures and images for: Building Memory Representations for Exemplar-Based Judgment: A Role for Ventral Precuneus
Source: Front Hum Neurosci. 2019 Jul 16;13:228. doi: 10.3389/fnhum.2019.00228 (PMC6646524; doi:10.3389/fnhum.2019.00228)

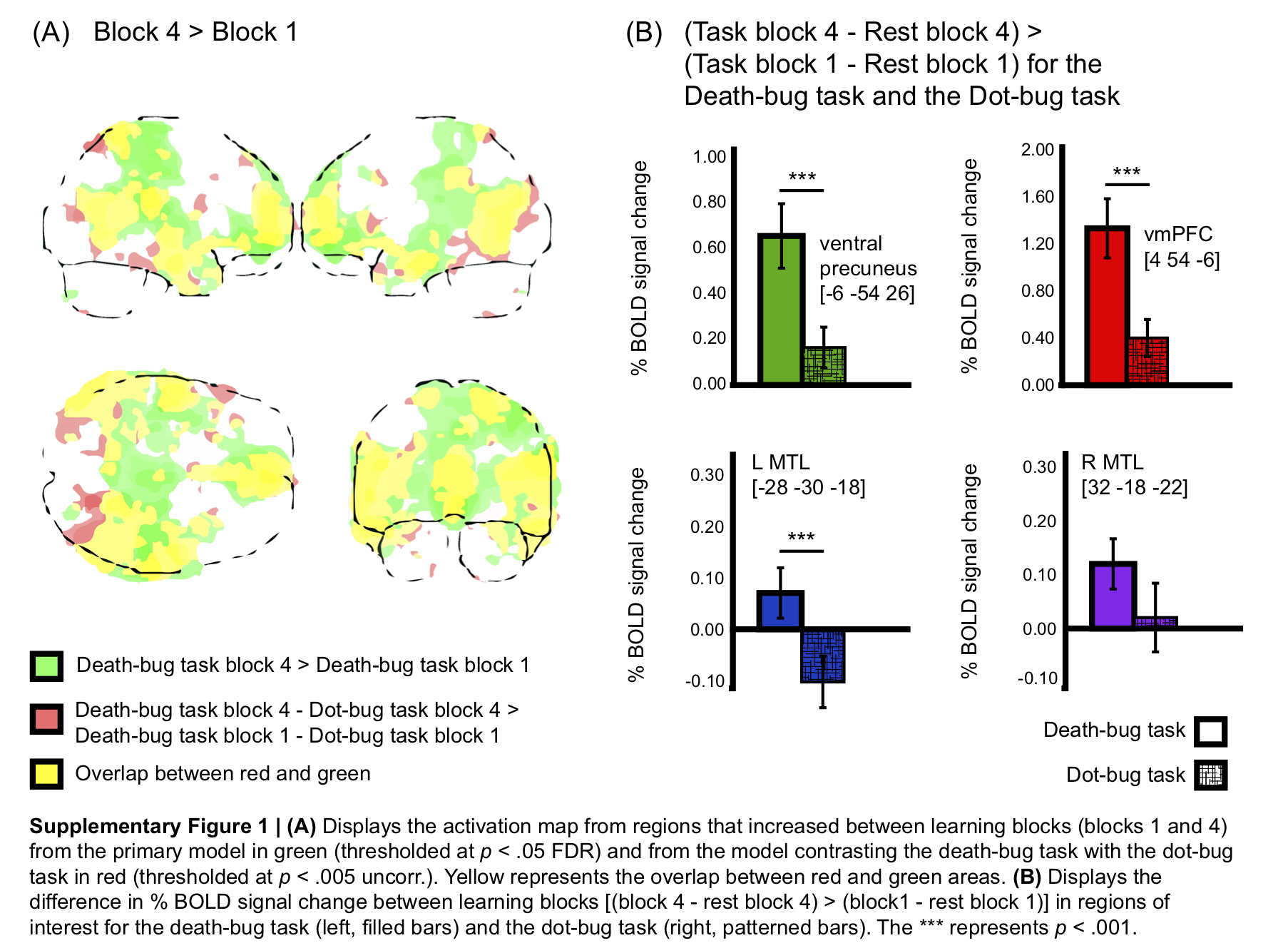

Supplement: Supplementary file 1 [file Image_1.jpeg]
